# A Proposed Trial Design for the Treatment of Widely Metastatic Ewing Sarcoma Inspired by Evolutionary Dynamics

**DOI:** 10.3390/cancers14030736

**Published:** 2022-01-31

**Authors:** Jonathan Metts, Thomas Russell, Damon Reed, Matteo Trucco

**Affiliations:** 1 Cancer and Blood Disorders Institute, Johns Hopkins All Children’s Hospital, St. Petersburg, FL 33701, USA; 2 Departments of Pediatrics, Wake Forest University School of Medicine, Winston-Salem, NC 27157, USA; trussell@wakehealth.edu; 3 Moffitt Cancer Center, Department of Individualized Cancer Management, Tampa, FL 33612, USA; damon.reed@moffitt.org; 4 Department of Pediatric Hematology Oncology and BMT, Cleveland Clinic Children’s, Cleveland, OH 44106, USA; truccom@ccf.org

**Keywords:** Ewing sarcoma, clinical trial, evolutionary dynamics

## Abstract

**Simple Summary:**

Metastatic Ewing sarcoma requires novel strategies to improve dismal cure rates. We propose a clinical trial based on the application of evolutionary oncology principles to Ewing sarcoma.

**Abstract:**

Metastatic Ewing sarcoma has dismal long-term survival despite multiple attempts to intensify standard therapy through the addition of new agents to the existing chemotherapy backbone. Here, based on the application of evolutionary dynamics to pediatric sarcoma, we propose an alternative treatment strategy that varies exposure to agents and dosing intensities, termed sequential second-strike therapy (SSST). We announce an upcoming clinical trial to apply these principles to patients with widely metastatic Ewing sarcoma, those with metastatic disease beyond the lungs.

## 1. Introduction

Ewing sarcoma (ES) is the second most common bone cancer in children and adolescents. Systemic chemotherapy is standard alongside local control; however, improvements in outcomes in recent decades due to chemotherapy are mainly seen in localized ES (LES), without clear improvements in outcome in metastatic ES (MES). Cooperative group studies IESS-MD I and II demonstrated an initial response rate of 70–73% to intensive chemotherapy in MES, but only 28–30% 3-year event-free survival (EFS) was observed [1]. The disparity between initial response and long-term outcome is consistent with a model of a minor, treatment-resistant population of ES present prior to or developing during systemic therapy that leads to relapse. This suggests that changing therapy at some point to eradicate this resistant population is needed to prevent relapse; an eco-evolutionary approach [2,3]. Many mechanisms of resistance can be hypothesized, each requiring different strategies to overcome them. These include, but are not limited to, resistance to therapy due to cell cycling that is not aligned with every 2- to 3-week chemotherapy cycle requiring altered scheduling, limited drug exposure requiring higher chemotherapy dosing, cell dormancy causing very rare cycling and requiring more protracted exposure to therapy, or rapid cancer cell evolution during therapy exposure [4]. As there are likely multiple mechanisms of resistance occurring simultaneously in MES, adding any one of these strategies alone to standard therapy would likely not succeed, but a sequence of them may. 

While the addition of ifosfamide and etoposide (IE) to vincristine, doxorubicin, and cyclophosphamide (VDC) led to a significant improvement in the 5-year EFS in LES from 54% to 69%, no benefit was seen in MES, with 5-year EFS rates of 22% regardless of regimen [5]. Multiple attempts to add to the VDC (VDC/IE) backbone for MES, including the addition of an anti-angiogenic regimen with vinblastine and celecoxib or addition of the anti-IGFR antibody ganitumab, have failed to improve outcomes [6,7]. Even high-dose chemotherapy with autologous stem cell rescue for MES patients has been controversial at best, with increased toxicity and an indeterminate effect on survival without a clear signal in the metastatic population [8,9]. These results, coupled with the lack of benefit in LES with the addition of topotecan, suggest that response rates in the relapsed population are not a reliable tool to predict agents that can improve survival in newly diagnosed patients [10,11,12]. While repetitive use of active combination chemotherapy at maximum tolerated doses (MTD) is clearly beneficial for LES, these benefits appear minimal or non-existent for MES. Thus, the rationale for continued attempts to layer novel agents or “treatment cassettes” onto the VDC/IE backbone in MES must be questioned. 

We thus propose to extend to MES a previously reported conceptual framework of treating pediatric sarcomas with evolutionary-inspired strategies [3]. This is illustrated practically by an active clinical trial for patients with metastatic, fusion-positive rhabdomyosarcoma (NCT04388839). Instead of repeating the same intensive chemotherapy every 2 or 3 weeks, we will alter chemotherapy drugs, schedule, and intensity through a series of “second-strikes” (SS) to address resistant ES cells with multiple strategies. In evolutionary terms, our therapeutic goal is to drive the population numbers from no evidence of disease (NED) radiographically to below the minimum viable population (MVP), an ecological term whereby population numbers are low enough and fragmented to more reliably lead to extinction (cure of MES). A similar strategy of altering chemotherapy combinations and intensities, serendipitously developed over several decades, has resulted in excellent cure rates for pediatric acute lymphoblastic leukemia (ALL) [13].

## 2. Materials and Methods

This concept began in November 2020 with recurring teleconferences between investigators of the Sunshine Project, a multi-institutional clinical trial consortium, where we discussed alternative trial strategies for poor-prognosis sarcomas. We initially developed consensus on principles to guide this trial’s development, which were:

- We are not “one drug away” from significantly improving outcomes for MES.
- After initial treatment (the “first-strike”; FS), therapy should be altered by changing agents and intensities at frequent intervals to affect small populations of resistant ES, here referred to as sequential second-strike therapies (SSST).
- A prolonged and varied maintenance phase may be beneficial to drive the cancer cell population from a radiographic NED to below the MVP.
- We should incorporate biological and radiomic correlates over time, including circulating tumor cells and DNA (CTC and ctDNA) prior to every change in systemic therapy.

The trial strategy was then reviewed and critiqued in multiple teleconferences and larger investigator meetings, including the National Pediatric Cancer Foundation’s Sunshine Project retreats in February and August of 2021 before final consensus on treatment strategy was obtained.

## 3. Results

We propose the following regimen in a feasibility study focused on patients with the poorest prognosis of MES, those with metastatic disease beyond the lung (widely metastatic Ewing Sarcoma, WMES). Both children and adults will be eligible for this trial without predefined age limits. We used the prognostic scoring tool developed by Ladenstein et al. to define our target patient population (Ladenstein score >3) with a 3-year EFS of ≤25% [8]. Each agent incorporated in this proposed trial was used with regularity for the treatment of ES, albeit in different combinations, in relapsed settings, and or in trials not exclusive to ES. The objectives of this trial are to establish the feasibility and tolerability of an SSST approach to WMES, obtain sequential biologic and radiomic correlates to study the biology of WMES throughout treatment, and obtain preliminary data on the effect of SSST on the event-free and overall survival of patients with WMES. This trial will also serve as an opportunity to collect prospective data on “Ewing-like” sarcomas. Due to the rarity of diagnosis, a lack of standardized treatment approaches, and early data suggesting equivalent or inferior prognosis to Ewing sarcoma depending on translocation type, we also plan to enroll patients with metastatic Ewing-like sarcomas with BCOR- and CIC- rearrangements on a separate stratum [14].

**Figure 1:**
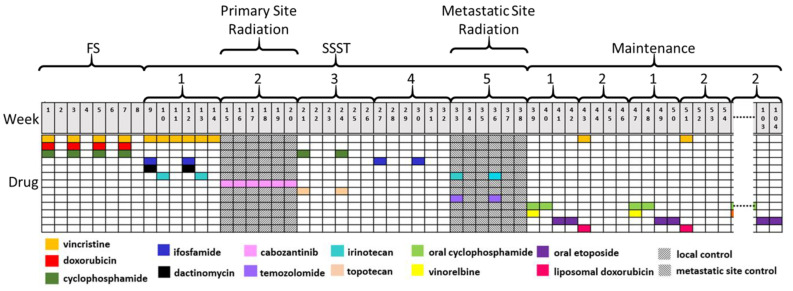
Treatment schema of sequential second-strike therapy for widely metastatic Ewing sarcoma. After four cycles of vincristine, doxorubicin, and cyclophosphamide, therapy is altered at 6-week intervals between unique combinations of varying intensity before entering a prolonged maintenance phase. Abbreviations: FS; first-strike, SSST; sequential second-strike therapy.

The proposed regimen (Figure 1, Table 1) begins with an FS intended to reduce the bulk or tumor cells using VDC. Given the equivalence of VDC and VDC/IE in MES, we chose to give the VDC in an intensive, “interval compressed” schedule every two weeks for four cycles [5]. We then changed therapy to the first of many SS: two cycles of a more novel combination of chemotherapies, IrIVA [15,16,17]. This attempts to address cells that resist therapy due to entering the S phase after the initial day 1 ifosfamide/vincristine/actinomycin insult. This was accomplished by administering irinotecan on days 8–12, as demonstrated recently by Ferrari et al. in ES and other sarcomas. This schedule slightly delays the timing of local control from the conventional week 12 to week 15. Data from the National Cancer Database suggest better outcomes for LES patients who begin local control by week 15 vs. week 16 or beyond; therefore, it is felt this delay will not be detrimental [18]. 

We then shifted focus to local control of the primary site with radiation. During primary site radiation, we continued, but lessened the intensity of, systemic therapy through the next SS with a tyrosine kinase inhibitor (TKI) administered orally continuously for six weeks. We proposed cabozantinib administered in recent studies showed activity against ES and safety data when combined with radiation [19,20].

After radiation, we entered a third SS, introducing topotecan and cyclophosphamide, an established combination for relapsed ES, for two 21-day cycles [10,21]. The fourth SS, two 21-day cycles of high-dose ifosfamide at 15 g per cycle, again increased the intensity and addressed resistance that may be overcome with higher dosing based on preliminary results in the ongoing rEECur trial [22]. The fifth SS, two 21-day cycles of irinotecan and temozolomide, were combined with radiation therapy addressing the metastatic sites of disease. Both irinotecan and temozolomide have evidence of radiosensitization in other cancers (including GI and CNS), were used safely in combination with radiation for several sarcomas, and are a standard regimen for relapsed ES [23,24,25,26,27]. Up to this point, after the initial VDC “induction”, this trial regularly altered therapy every 6 weeks, never returning to a previously-received drug combination, though revisiting some alkylators in new combinations. 

Upon completion of the therapy above, it is believed that bulk disease was thoroughly addressed with the combinations of chemotherapeutic agents and radiation to the primary and metastatic sites, rendering the patient radiographically NED. The patient then began a maintenance phase intended to tackle dormant or rarely cycling “minimally residual disease”. This phase involved two additional SS, each lasting 28 days, that alternated for approximately 18 months. This first maintenance SS included vinorelbine and oral cyclophosphamide for two weeks followed by oral etoposide for two weeks. The second maintenance SS used vincristine and liposomal doxorubicin administered on day 1 of the 28-day cycle. These two SS continued to alternate until the end of maintenance. Much like ALL, dose reductions and breaks in the maintenance phase were anticipated and were guided by the protocol to facilitate tolerability. 

## 4. Discussion

Upon initial visualization, the proposed strategy may appear overwhelmingly toxic. However, an evaluation of the cumulative dosing compared to a current standard, AEWS0031, suggests otherwise (Table 2). First, it must be noted that the treatment in the proposed trial is administered over a two-year (104-week) period, as opposed to 29 or 42 weeks for AEWS0031. This proposed treatment has a total cumulative cyclophosphamide equivalent dose that is 3 g/m^2^ lower than AEWS0031, and the etoposide dose is approximately two-thirds of that administered in AEWS0031 [28]. While anthracycline dosing is higher, the 375 mg/m^2^ conventional doxorubicin will be administered with the cardio-protectant medication dexrazoxane, and the remaining 240 mg/m^2^ will be administered in the form of liposomal doxorubicin. Furthermore, chemotherapy with more favorable side effect profiles is used during maintenance, adopting oral or liposomal formulations when possible. The regimens, schedules, and combinations proposed were, however, constructed with keen attention to toxicity and a conscious effort to vary the intensity of the phases and attempt to maximize the tolerability of the treatment as a whole. For example, most of the treatments outlined here are intended for the outpatient setting with standard administration. Accommodations will be incorporated into the protocol, and treating physicians will be given latitude with the use of supportive measures.

This proposed trial is also designed to evaluate potential biomarkers of therapeutic response at frequent intervals when chemotherapy regimens change, approximately every 6 weeks. Past and ongoing studies to evaluate CTC and ctDNA in sarcomas hint at the value of these techniques [29,30,31]. Specifically, single-cell RNA sequencing on CTC will be important to observe the evolution of ES cells as these sequential therapies are applied. The addition of these evaluations, combined with periodic imaging, including radiomic evaluation, can shed light on the effect of the various chemotherapy combinations, schedules, and intensities on the overall response seen in the enrolled patients. These serial evaluations, in addition to molecular analysis of tumor biopsies, may identify specific prognostic strata and help tailor future sequential therapies. Furthermore, any evidence generated by this trial that an approach using sequential SS of chemotherapy would improve outcomes may in turn influence preclinical testing of drugs. Instead of testing single agents or combinations at a maximum tolerated dose, in vitro and in vivo testing could aim to evaluate and optimize what sequence of treatments would maximize response [32].

## 5. Conclusions

The proposed clinical trial treats WMES with SSST designed to drive the number of ES cells below the MVP necessary to cause relapsed and recurrent disease. This strategy uses agents typically accepted for the treatment of ES and administers them in a sequence and schedule that increases the number of days the cancer is exposed to chemotherapy and in a manner that changes drug combinations and schedules every few weeks. The treatment is also protracted and involves both initial and delayed intensive strikes guided by the past clinical trial data, as well as less intensive but persistent strikes. While daunting, we believe treatment with SSST is tenable and hypothesize that MES is at least as challenging to cure as ALL and thus, a similar duration and variation of agents and schedules would be necessary to begin to approximate a similar level of efficacy. We are aware that the specific interventions that lead to any improvement detected would later need to be discerned, though the valuable correlates built into this trial will provide some insight. This strategy will also benefit from, and hopefully inspire, subsequent preclinical testing, shifting focus from reducing established tumors to eradicating residual cancer cells after initial debulking treatments or preventing recurrence in other models of minimally residual disease. Expanded trials would also study and test this treatment approach more accurately once feasibility is established. 

## Figures and Tables

**Table 1:** Chemotherapy regimens with doses used in the sequential second-strike trial for widely metastatic Ewing sarcoma. Abbreviations: FS; first-strike, SSST; sequential second-strike therapy.

	FS	SSST					Maintenance	
		1	2	3	4	5	1	2
**Regimen and Doses**	Vincristine 1.5 mg/m^2^ Day 1 Cyclophosphamide 1200 mg/m^2^ Day 1 Doxorubicin 75 mg/m^2^ Day 1	Ifosfamide 3 g/m^2^ Days 1,2 Vincristine 1.5 mg/m^2^ Days 1,8,15 Actinomycin 1.5 mg/m^2^ Day 1 Irinotecan 20 mg/m^2^ Days 8–12	Cabozantinib 40 mg/m^2^/day (60 mg/day patients 16 years and older)	Topotecan 0.75 mg/m^2^ Days 1–5 Cyclophosphamide 250 mg/m2 Days 1–5	Ifosfamide 3 g/m^2^, Days 1–5	Irinotecan 50 mg/m^2^ Days 1–5 Temozolomide 100 mg/m^2^ Days 1–5	Oral Cyclophosphamide 25 mg/m^2^ Days 1–14 Vinorelbine 25 mg/m^2^ Days 1 Oral Etoposide 40 mg/m^2^ Days 15–28	Vincristine 1.5 mg/m^2^ Day 1 Liposomal Doxorubicin 30 mg/m^2^ Day 1
**Length of Therapy**	14-day cycles, Four cycles total	21-day cycles Two cycles, total	42-day cycle, One cycle total	21-day cycles, Two cycles total	21-day cycles, Two cycles total	21-day cycles, Two cycles total	28-day cycles, Alternating with Maintenance 2 until 2 years	28-day cycles, Alternating with Maintenance 1 until 2 years

**Table 2:** Cumulative chemotherapy exposure on the sequential second-strike trial compared to standard treatment (AEWS0031 Regimen B) Abbreviations: CED; cyclophosphamide equivalent dose, SSST; sequential second-strike therapy.

	Cyclophosphamide	Ifosfamide	Doxorubicin	Etoposide
AEWS0031, regimen B (over 29 weeks)	8.4 g/m^2^	63 g/m^2^	375 mg/m^2^	3500 mg/m^2^
	Total CED: 23.8 g/m^2^			
SSST Protocol (over 104 weeks)	10.5 g/m^2^ (7.3 g/m^2^ IV, 3.2 g/m^2^ oral)	42 g/m^2^	540 mg/m^2^ (Includes 240 mg/m^2^ Liposomal)	2240 mg/m^2^ equivalent (4480 mg/m^2^ oral, 2:1 IV to oral equivalency)
	Total CED: 20.8 g/m^2^			

## Data Availability

The data presented in this study are available in this article.
